# Community-Level Factors Associated with COVID-19 Cases and Testing Equity in King County, Washington

**DOI:** 10.3390/ijerph17249516

**Published:** 2020-12-18

**Authors:** Edmund Seto, Esther Min, Carolyn Ingram, BJ Cummings, Stephanie A. Farquhar

**Affiliations:** 1 Department of Environmental & Occupational Health Sciences, University of Washington, Seattle, WA 98195, USA; eseto@uw.edu (E.S.); estmin@uw.edu (E.M.); bjcumngs@uw.edu (B.C.); 2 Bordeaux School of Public Health, University of Bordeaux, 33076 Bordeaux, France; carolyn.ingram@etu.u-bordeaux.fr; 3 Department of Health Services, University of Washington, Seattle, WA 98195, USA

**Keywords:** COVID-19 testing, disparities, health inequities, socioeconomic status, COVID-19 risk, COVID-19 positivity, environmental justice

## Abstract

Individual-level Coronavirus Disease 2019 (COVID-19) case data suggest that certain populations may be more impacted by the pandemic. However, few studies have considered the communities from which positive cases are prevalent, and the variations in testing rates between communities. In this study, we assessed community factors that were associated with COVID-19 testing and test positivity at the census tract level for the Seattle, King County, Washington region at the summer peak of infection in July 2020. Multivariate Poisson regression was used to estimate confirmed case counts, adjusted for testing numbers, which were associated with socioeconomic status (SES) indicators such as poverty, educational attainment, transportation cost, as well as with communities with high proportions of people of color. Multivariate models were also used to examine factors associated with testing rates, and found disparities in testing for communities of color and communities with transportation cost barriers. These results demonstrate the ability to identify tract-level indicators of COVID-19 risk and specific communities that are most vulnerable to COVID-19 infection, as well as highlight the ongoing need to ensure access to disease control resources, including information and education, testing, and future vaccination programs in low-SES and highly diverse communities.

## 1. Introduction

The first confirmed case of Coronavirus Disease 2019 (COVID-19) in the US was found in Washington state in a community north of Seattle on 21 January 2020. Shortly afterwards, on 19 February, an outbreak of cases within a nursing home was identified. Subsequent to these early cases, COVID-19 was declared a global pandemic, and transmission had spread throughout the US. By 12 July 2020, 41,748 confirmed cases had been identified in Washington state, with 29% (12,131) of the cases occurring in the Seattle, King County area [1].

While COVID-19 affects all populations, it has impacted some more than others. Within Washington, analyses of confirmed case, hospitalization, and mortality data have found that communities of color are disproportionately impacted by COVID-19 [2,3]. Specifically, the highest age-adjusted case, hospitalization, and death rates are observed among Hispanic and Native Hawaiian and other Pacific Islander populations (NHOPI) (Hispanics 2990.9 cases per 100,000, 322.2 hospitalizations per 100,000, and 101.6 deaths per 100,000; NHOPI 2940.8 cases per 100,000, 551.9 hospitalizations per 100,000, and 134.8 deaths per 100,000). Understanding these demographic trends is critical to ongoing targeted outreach efforts to reduce disease transmission.

Communities of color in the United States face a combination of structural, social, and environmental inequities that increase risk for infection and severe illness [4]. Generally, race and socioeconomic status are two primary contributors to health disparities, including access to healthcare and affordable comprehensive insurance [5,6]. In addition, people of color are more likely to work in essential jobs, live in overcrowded housing conditions, and use public transportation [4], elevating the risk of exposure to COVID-19. Testing sites in major US cities have reportedly been concentrated in predominantly white areas [7,8]. In New York City, the number of distributed tests increased with the proportion of white residents per zip code through April 2020 [7]. In Illinois, Black residents, making up 37% of the state’s COVID-19 deaths, had only received 13% of the state’s tests [9], and similar patterns emerged across Texas [8].

Interventions targeting social determinants of health have proven to be effective in improving health outcomes of individuals and communities [10,11]. However, few studies have considered community-level social and built environmental factors such as socioeconomic status and race and their potential role as upstream determinants of COVID-19 infection. Because certain activities, such as provision of testing services, are geographic in nature and neighborhood focused, analyses of community-level factors may provide added insight into the ongoing disease control needs [10,12]. We hypothesize that low socioeconomic conditions, language barriers, and limited access to testing may be important impediments to identifying cases in communities [10].

One community-focused study has been conducted for Massachusetts [13], which found higher rates of COVID-19 cases with increasing percentages of Black and Latino populations within communities. The authors of the study noted that these community-level health disparities may exist for various reasons, including a potentially greater participation of certain population groups in essential work, differences in housing density, propensity to live in multigenerational households, barriers to healthcare faced by foreign born non-citizens, and further upstream historical inequities that have resulted in differential access to care. Moreover, analyses of COVID-19 in major urban areas in the US identified poverty as a major factor that is associated with confirmed cases and deaths [14].

Furthermore, few studies have considered the community-level factors that may be associated with COVID-19 testing. However, some evidence is emerging on the importance of testing. For example, comparing statistics for different countries has found an association between higher testing rates and lower mortality rates [15]. Within the US, the relationship between the numbers of COVID-19 tests and numbers of identified cases has been somewhat controversial [16]. Only one study to date has analyzed the associations between population race, ethnicity, or socioeconomic factors and rates of testing within communities [7].

The goal of this study is to assess the relationships between community-level indicators including poverty, educational attainment, linguistic isolation, and racial and ethnic composition in COVID-19 testing and case rates in the Seattle, King County, Washington region.

## 2. Methods

### 2.1. Measures: King County, WA COVID-19 Testing Data, and Census Tract-Level Data

Data on the numbers of COVID-19 tests and confirmed cases (positive tests) by residential census tract as of 12 July 2020 were obtained from the Public Health Seattle & King County (PHSKC) COVID-19 data dashboard. These testing data corresponded to peak infection in the county during the summer of 2020. The first peak in the numbers of positive cases occurred between March and June, while a second peak occurred from July, reaching highest levels in mid-July, and then falling through August and September. The county defines “tests” as the number of unique residents who have had a COVID-19 PCR laboratory result reported to the Washington State Department of Health (DOH). Therefore, residents who had more than one test are counted once. Testing rate is the count of tests in a census tract divided by the total population of residents in that census tract and reported as tests per 1000 residents. The county defines “positive cases” as the number of unique residents who have had a positive COVID-19 PCR laboratory result reported to DOH. Our analysis considers positive test rate as the count of positive tests in a census tract divided by the total number of unique resident tests reported for the tract. In all cases, the census tract in which the test taker permanently resides was used, rather than where the test was conducted.

Census tract-level data were obtained from the Washington State Department of Health’s Washington Tracking Network, which compiles community indicators based on the 5-year (2013–2017) American Community Survey for educational attainment (percent of people who have not received a high school diploma or General Educational Development (GED) by the age of 25), poverty (the percentage of people living under 185% of the federal poverty level), housing cost burden (the percentage of households spending > 30% of income on housing), transportation cost burden (the transportation costs based on percentage of income for the regional moderate household). The Center for Neighborhood Technology (CNT) defines regional moderate household income as a household income of 80% of the area median, the regional average household size, and the regional average commuters per household, unemployment percentage, linguistic isolation (percentage of the population five years and older that speak English less than “very well” and “not at all”). Race and ethnicity were considered in two different ways: first with a composite “people of color” (POC) indicator, representing the percentage of the population that is non-white, and second as disaggregated race/ethnicity composition (percentages of Hispanic, and non-Hispanic White, Black, American Indian/Alaskan Native, Asian, and Native Hawaiian/Pacific Islander), with the later analysis used to identify disproportionate impacts of COVID-19 on racial/ethnic populations.

### 2.2. Statistical Analyses

To assess the associations between COVID-19 test positivity and community-level indicators, multivariate generalized linear models (GLM) with a Poisson link function and robust standard errors were used to estimate the outcome of interest (the number of confirmed positive COVID-19 tests at the census tract level) based on various census tract-level indicators. All models included the natural logarithm of the number of COVID-19 tests on residents in each census tract as an offset variable. The first model assessed the association between COVID-19 test positivity and socioeconomic status (SES) indicators and the POC variable. The second model substituted the POC variable with separate race/ethnicity independent variables. Because of potential collinearity among the independent variables, Pearson correlations were used to identify highly correlated variables (>0.80), with one of the highly correlated variables removed from the set of independent variables in the Poisson regression model as described in the results below. The spatial autocorrelation of the model residuals was tested using the Moran’s I statistic. For model prediction, spatially autocorrelated residuals were modeled using a spherical variogram, and kriged residuals were added to the GLM estimates. Similarly, Poisson GLMs were applied to testing rate data, with the outcome of interest (the number of COVID tests conducted in each tract), offset by natural logarithm of the census tract population. Geographically weighted regression (GWR) using the Poisson model was conducted for both outcomes of interest (case positivity and test rate outcomes) with a neighborhood search on the optical number of neighbors and Akaike information criterion (AIC) for model selection. All analyses were conducted in R version 3.6.2 (R Foundation for Statistical Computing, Vienna, Austria), with robust standard errors estimated by the Sandwich 2.5–1 package, with the exception of GWR, which was conducted in ArcGIS Pro 2.6.0 (Esri Inc, Redlands, CA, United States)

## 3. Results

By 12 July 2020, a total of 208,546 tests for COVID-19 had been performed in King County. Of these, 11,599 were confirmed to be positive cases, representing a positivity rate of 5.6%. Summary statistics for census tract-level indicators are presented in Table 1, grouped by quartile of the number of confirmed cases per census tract. Trends can be observed in a number of indicator variables. For example, the mean number of tests per tract increases with the number of confirmed cases per tract. Many of the SES variables indicate the trend relationship between low-SES and higher numbers of confirmed cases per tract. There is also a trend with an increasing proportion of POC residents and higher positive COVID-19 cases. Tracts with higher percentages of Hispanic, Black, and Asian populations tend to have higher numbers of COVID-19 cases. However, tracts with greater proportions of White residents tend to have lower case numbers.

**Figure 1:**
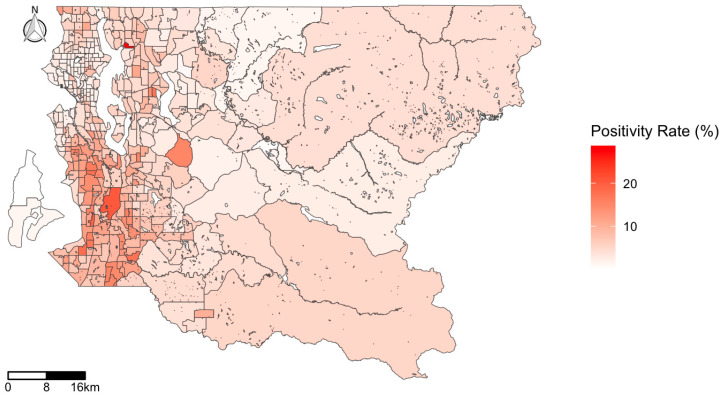
Map of COVID-19 test positivity rate (positives per test) by census tract in King County, WA, USA.

**Figure 2:**
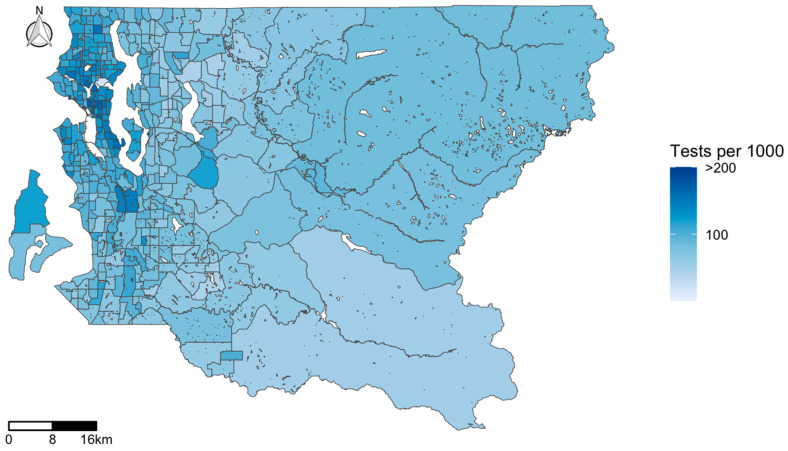
Map of COVID-19 testing (tests per 1000 population) by census tract in King County, WA, USA.

The map of the COVID-19 test positivity rate in King County (Figure 1) highlights the clustered nature of infection, with tracts located in the urban core of Western King County more highly impacted. While some individual tracts stand out as having considerably higher test positivity than their surrounding tracts (these were the sites of senior care centers with COVID-19 outbreaks), generally the southwestern region of the county has a high rate of infection. In contrast, the map of COVID-19 testing (Figure 2) illustrates high rates of testing clustered in the downtown area, with the highest testing rate of 575.5 per 1000 population in a single census tract in downtown Seattle. While testing rates are higher in the northwestern parts of Seattle, test positivity is concentrated in the southwestern part of the county.

Before assessing the association between community tract-level factors and COVID-19 cases, Pearson correlations were computed between the tract-level variables. Linguistic isolation and POC were found to be highly correlated (0.853), and thus the former was dropped from the multivariate models. Similarly, housing cost burden and poverty were found to be highly correlated (0.815), and thus the former was dropped from the models. For the race/ethnicity variables, percent White and percent Black were found to be inversely correlated (−0.711), and thus the former was dropped from the models.

**Figure 3:**
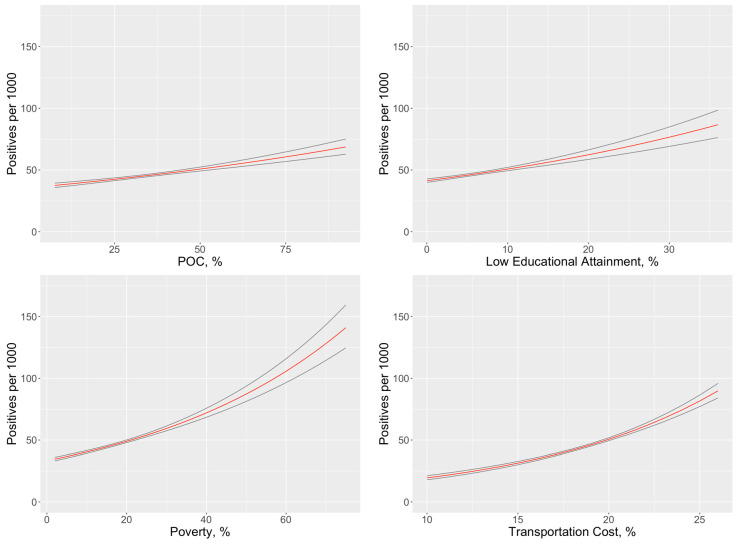
Influence of community-level factors on test positivity rates, adjusting for other factors in the multivariate model, with main estimate shown in black and the 95% CI in red.

The first regression model estimated the associations between the number of COVID-19 cases (adjusted for testing numbers) and tract-level SES variables and the POC variable (Table 2). SES variables, including census tract-level poverty and census tracts with residents who spend a higher proportion of their income on transportation cost were found to be significantly associated with higher numbers of COVID-19 cases. Adjusted for SES, tracts with higher proportions of POC residents were also associated with higher numbers of COVID-19 cases. The modeled effect of each variable may be observed in Figure 3, with other covariates set at median levels. Higher levels of each of the variables included in the model are associated with higher test positivity rates, with community-level poverty having a strong influence on positivity rates.

The second regression model estimated the associations between the number of COVID-19 cases (adjusted for number of tests) and tract-level SES variables and separate race/ethnicity variables (Table 3). As with the previous model, the tracts with low-SES conditions were associated with higher numbers of COVID-19 cases. Of the race/ethnicity variables, after adjustment for SES conditions, the percentage of residents in the tract that are Hispanic was associated with the greatest increase in numbers of COVID-19 cases per tract, with a 3.0% increase in COVID-19 cases with each percentage increase in Hispanic population. The next largest increase was found for Black or Asian residents, with a 1.1% increase in COVID-19 cases per tract with each percentage increase in Black or Asian population. Finally, the percentages of American Indian/Alaskan Native or Native Hawaiian and Pacific Islander residents were not found to be significantly associated with COVID-19 case numbers; however, the lack of statistical significance may be due to smaller population compared to other racial and ethnic groups.

**Figure 4:**
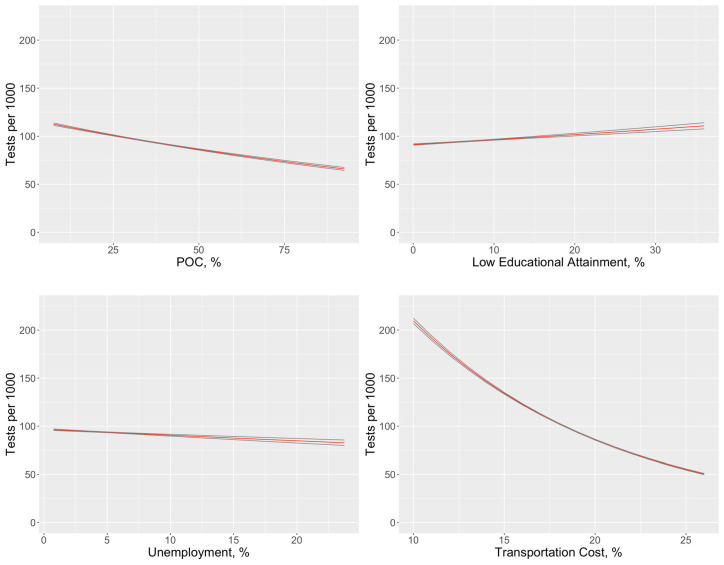
Influence of community-level factors on testing rates, adjusting for other factors in the multivariate model, with main estimate shown in black and the 95% CI in red.

To examine the factors related to COVID testing rates, a third regression model estimated the associations between COVID tests (adjusted for tract population) and tract-level SES variables and the POC variable (Table 4). SES factors were less pronounced in these results than those observed for COVID test positivity (Table 4 vs. Table 2). However, communities with higher proportions of POC tended to have lower testing rates. Additionally, communities with higher transportation cost burden tended to have lower testing rates. The modeled effect of each variable may be observed in Figure 4, with other covariates set at median levels. The strong influence of transportation cost can be clearly seen as a barrier to testing.

A fourth model, which separated out the POC variable into distinct variables for proportions of racial and ethnic categories, provided additional information on the factors associated with testing rates by community (Table 5). Hispanic communities, despite having the highest test positivity rates, had lower testing rates than other communities. Asian and Native Hawaiian/Pacific Islander communities also had statistically significant lower rates of testing than other communities. Moreover, with respect to socioeconomic barriers to testing, communities with high transportation cost burdens were found to have lower testing rates. Interestingly, communities with lower educational attainment tended to have higher rates of COVID testing.

Geographically Weighted Regression maps are presented in Supplementary Materials. Firstly, two GWR models for test positivity were explored. These spatial models had the same set of independent variables as their corresponding non-spatial Poisson models previously presented in Table 2 and Table 3. The simpler GWR model with only the people of color variable instead of the separate race/ethnicity variables was found to produce a lower AIC (390.3 vs. 418.5). This model was found to explain 72.9% of the deviance in COVID-19 test positivity. The Moran’s I of the model’s deviation residuals was 0.00093, and not significantly different from the expected Moran’s I for spatial randomness (*p* < 0.61). Coefficient maps for each of the explanatory variables are shown in Supplementary Materials, Figure S1. Low educational attainment was associated with higher rates of COVID-19 in neighborhoods at the top of Lake Washington and in the Issaquah area. Poverty was associated with higher rates in the area above Lake Sammamish. The relationships between the people of color variable and COVID-19 rates were observed in several areas in the eastern portion of the county, such as in the Bellevue-Kirkland, Northgate, Vashon Island, and Federal Way areas. Additionally, transportation costs were associated with higher positivity rates in the northern part of the county.

Secondly, two GWR models for testing rate were explored. These spatial models had the same set of independent variables as their corresponding non-spatial Poisson models previously presented in Table 4 and Table 5. The simpler GWR model with only the people of color variable instead of the separate race/ethnicity variables was found to produce a lower AIC (1344.3 vs. 1347.0). This model was found to explain 83.7% of the deviance in COVID-19 testing rates, yet slight spatial autocorrelation was still observed in the Moran’s I of the deviation residuals (−0.020, *p* < 0.0092). Coefficient maps for each of the explanatory variables are shown in Figure S2, and are distinct from the spatial patterns observed for test positivity. Low educational attainment was associated with lower testing rates, particularly in the area near Lake Union. Unemployment was associated with lower testing rates in various regions, including the Lake Vashon, Federal Way, Kirkland, and Woodinville-Novelty Hill areas. The people of color variable was associated with lower testing rates in the Seattle downtown area. Transportation costs were associated with lower testing in West Seattle and surprisingly in the Seattle downtown area. The issue of transportation cost (high proportion of income spent on transportation) in downtown Seattle may reflect poverty among some populations in the downtown area.

## 4. Discussion

This is one of the few studies that has assessed the association between community-level indicators and COVID-19 testing rates and test positivity. Relationships were observed between census tracts with higher numbers of tests conducted, lower SES, and higher percentages of POC residents and higher rates of confirmed COVID-19 cases. Multivariate models that adjusted for variations in the amount of testing by census tract found significant associations between the rate of COVID-19 cases per tract and tract-level SES variables including poverty, educational attainment, and income spent on transportation cost. Higher rates of COVID-19 positive cases were also observed for tracts with higher proportions of POC residents, Hispanic residents especially, and to a lesser extent, Black and Asian residents. It is important to note that these significant associations were identified with Poisson models that included the number of unique persons tested as an offset variable, and thus control for varying amounts of testing conducted in each tract.

Testing rates were also found to be associated with census tract-level factors. Tracts with higher proportions of POC residents, greater unemployment, and higher proportions of income spent on transportation costs tended to have lower rates of COVID-19 testing. Despite Hispanic communities having the highest rates of positive tests, they had the lowest testing rates. In New York City, similar testing disparities were observed in the early months of the pandemic. Zip codes with a greater proportion of white residents had a higher number of COVID tests [7].

In addition, test positivity was closely associated with race/ethnicity and SES. Maps of the COVID-19 test positivity identified southwest King County as having higher infection rates than other areas in the region. This part of the county has relatively lower SES and larger proportions of POC residents. There were clear differences in the spatial distribution of test positivity versus testing rates, with higher rates of positive tests identified in the southwestern portion of the county, but higher rates of testing conducted in the northwestern part of the county. Our findings are consistent with other studies. A national study found an association between COVID-19 positive cases and a community’s social vulnerability related to race, socioeconomic status, household composition, and housing and transportation [17]. In New York City, zip codes with lower SES experienced higher positivity [16].

Identifying these community-level factors is important for ongoing targeting of COVID-19 disease control resources. For example, earlier in the outbreak, access to public testing was limited to a few sites. Notably, the City of Seattle only offered one northern testing site on Aurora Avenue North and one southern testing site in a district south of downtown, the SODO district. While private providers and mobile clinics helped to fill in the gaps in spatial coverage, the city also started to offer new testing sites in the lower income, more ethnically diverse southern part of the city. This highlights the dynamic nature of COVID-19 infections and the ability of the regional public health agencies to respond quickly to these dynamics.

The associations between communities of color and testing rates and test positivity highlight the need for ongoing culturally tailored approaches to COVID-19 control strategies. Linguistic isolation was strongly correlated with the proportions of residents in a census tract that are people of color. Because of this correlation, we chose to only include POC in the multivariate models, but linguistic isolation is a suitable surrogate in our analyses. More research is needed to determine whether language barriers are actually responsible for disparities in testing and test positivity. It is worth noting though that already, PHSKC provides material and outreach in over 35 different languages. Moreover, there may be other correlating factors for POC and linguistically isolated populations, including barriers to healthcare access, potential stress and risk from essential work, and living in higher density households.

While we observed significant tract-level associations between community factors and COVID-19 testing and test positivity, there are likely other community-level factors at play that we have not yet analyzed. Some of these data are more difficult to obtain, but the underlying health status and access to care of a community may be important. Other stressors, such as food insecurity, job loss, assisting family and community members infected with COVID-19, and changing childcare needs all potentially operate at individual, family, household, and community levels to create pressures on COVID-19 testing and risks of acquiring infection.

Interestingly, the proportion of community residents spending high amounts of income on transportation costs was an important factor for both testing rates and test positivity. As previously mentioned, testing initially was limited to a few locations, and while additional testing centers have been introduced to target certain areas, transportation access seems to be an important barrier to COVID-19 control. If those most at risk for COVID-19 have limited access to transportation, they may be less likely to be tested, and if infections are not efficiently identified, ongoing transmission within communities may manifest as higher test positivity rates. Throughout the pandemic, access to and policies regarding public transit have evolved. For example, transit service was reduced due to lower ridership from stay-at-home orders; fare enforcement stopped for a while to reduce person-to-person contacts; and mask-wearing, sanitation, and physical barriers were implemented—all potentially creating uncertainties and stress, which may act as barriers to COVID testing. In addition to socioeconomic barriers, further research needs to be conducted to identify other barriers to testing. For example, essential workers living in southwest King County may have less time to access testing sites that are not conveniently located through or near places of employment.

The GWR results indicate that the associations between explanatory variables and COVID-19 positivity or testing rate are not uniform across geography, but that certain population factors may be more associated with either the high case rate or low testing rates in different parts of the county. Given the differences in the spatial distributions between case positivity and testing rates, it is not surprising that the maps of the underlying explanatory variable coefficients (Figures S1 and S2) are not the same. For example, the people of color variable was associated with lower testing primarily in the Seattle downtown area, whereas it was associated with higher case positivity in four regions outside of downtown, but surrounding Lake Washington. While there was no significant residual spatial autocorrelation in the GWR model for the case positivity model, there was slight remaining residual autocorrelation in the model for testing rates, which suggests that other community-level factors may be needed to better characterize the spatial pattern in testing.

By design, our study focused on community-level factors that may be associated with COVID testing and test positivity. Although ecological in nature, it points to important community factors that should be considered in individual-level studies of COVID-19 risk. As a next step, our group is conducting outreach to community members to further examine potential promoters and barriers to COVID-19 information, testing, future vaccination programs, and pandemic-related socioeconomic stressors using both qualitative and quantitative methods.

We engaged staff at PHSKC at the beginning of the project and shared our methods and research questions. Throughout the analysis, our team provided updates on our study as it was underway including preliminary results and interpretation of those results. The study team also met with local community organizations and multi-agency partnerships to discuss study results and solicit input on draft recommendations. Findings were distributed through virtual Zoom presentations, a PowerPoint presentation, a one-page study summary sheet, and a community fact sheet developed in consultation with community stakeholders and translated into Spanish. In addition, our results were shared through social media and via local media such as online newspaper and public television programming

Collectively, our findings may help inform ongoing efforts to prevent the spread of SARS-CoV-2 in the region. First and foremost, the maps highlight the need for continued access to disease control resources in the southwestern region of the county. In addition, because many of the communities with the largest numbers of test positives are in Hispanic, Black, and Asian neighborhoods, and include many people who are recent immigrants to the US, multilingual and culturally-appropriate outreach, health education, access to information and testing, and authentic community engagement by trusted community-based organizations and representatives are important considerations for effective disease prevention. Moreover, because higher numbers of COVID-19 cases are presenting from low-SES communities, it is critical that efforts be made to ensure that transportation costs, testing fees, and lack of medical insurance do not pose barriers to healthcare access and public health services. While the current study focuses on testing and test positivity data, as vaccines against COVID-19 are becoming available it will be important to assess the role of the community-level factors identified in this study that may affect the equitable delivery of vaccination programs.

## 5. Conclusions

This study assessed the association between community-level indicators and COVID-19 testing rates and test positivity. We found significant associations between the rate of COVID-19 cases per tract and tract-level SES variables including poverty, educational attainment, and income spent on transportation cost. These results demonstrate the ability to identify tract-level indicators of COVID-19 risk, and specific communities that are most vulnerable to COVID-19 infection, as well as highlight the ongoing need to ensure access to disease control resources, such as information and education, testing, and future vaccination programs in low-SES and highly diverse communities.

## Figures and Tables

**Table 1:** Summary statistics of census tract-level factors by quartiles of numbers of confirmed COVID-19 tests.

	Number of COVID-19 Cases <14			Number of COVID-19 Cases 14–22			Number of COVID-19 Cases 23–37			Number of COVID-19 Cases >37		
	Mean	Median	SD	Mean	Median	SD	Mean	Median	SD	Mean	Median	SD
Number of Tests	401.8	361.5	168.1	504.2	455.0	199.6	563.2	472.0	361.3	619.0	576.0	247.7
Low Educational Attainment (%)	3.6	2.4	3.5	5.0	3.7	4.6	8.1	6.0	6.7	13.6	12.8	7.7
Below 185% Federal Poverty (%)	12.4	10.4	9.1	15.1	13.1	9.5	20.8	18.3	11.1	32.1	30.3	13.1
Unemployment (%)	4.3	3.8	2.8	4.4	4.1	2.0	5.2	4.6	2.4	6.3	6.0	2.7
Linguistic Isolation (%)	5.0	3.5	5.2	7.6	5.9	6.7	11.7	10.3	7.7	17.0	15.5	8.6
Housing Cost Burdened (%)	28.7	27.6	7.9	30.7	29.9	7.0	34.7	34.5	8.0	40.1	39.9	7.9
Transportation Cost (%)	20.3	20.0	2.6	19.0	19.0	3.0	18.5	19.0	2.6	18.2	18.0	1.9
People of Color (%)	24.8	20.9	12.9	33.4	30.9	13.4	42.3	41.0	15.9	52.4	53.7	17.3
Hispanic (%)	5.1	4.7	2.9	6.4	5.8	3.4	10.1	8.4	6.6	15.5	14.7	9.7
White (%)	74.6	78.0	14.1	66.8	68.7	13.2	57.8	58.3	16.3	46.9	46.5	16.0
Black (%)	1.9	1.1	3.0	3.6	1.9	4.7	7.0	5.0	6.8	11.2	9.5	9.0
American Indian/Alaskan Native (%)	0.2	0.0	0.4	0.6	0.1	1.8	0.5	0.3	0.6	0.7	0.3	1.0
Asian (%)	13.2	8.6	13.0	16.5	13.6	11.5	18.3	16.1	10.8	18.0	15.6	10.2
Native Hawaiian/Pacific Islander (%)	0.2	0.0	0.5	0.5	0.0	1.7	0.9	0.0	1.6	1.5	0.3	2.5

**Table 2:** Results of multivariate model of COVID-19 test positivity based on socioeconomic status (SES) and people of color variables. Estimates and 95% CIs are the exponentiated coefficients of the Poisson model, with statistically significant associations indicated with an asterisk.

	Estimate	Robust SE	95% CI
Intercept	0.004	0.001	0.002–0.007
Low Educational Attainment (%)	1.021	0.006	1.009–1.032 *
Below 185% Federal Poverty (%)	1.019	0.003	1.014–1.025 *
People of Color (%)	1.007	0.002	1.003–1.012 *
Transportation Cost (%)	1.100	0.016	1.070–1.132 *

**Table 3:** Results of multivariate model of COVID-19 test positivity based on SES and race/ethnicity variables. Estimates and 95% CIs are the exponentiated coefficients of the Poisson model, with statistically significant associations indicated with an asterisk.

	Estimate	Robust SE	95% CI
Intercept	0.004	0.001	0.002–0.007
Low Educational Attainment (%)	1.005	0.006	0.993–1.016
Below 185% Federal Poverty (%)	1.015	0.003	1.010–1.021 *
Transportation Cost (%)	1.089	0.014	1.061–1.117 *
Hispanic (%)	1.030	0.004	1.021–1.038 *
Black (%)	1.011	0.004	1.003–1.019 *
American Indian/Alaskan Native (%)	1.005	0.017	0.973–1.039
Asian (%)	1.011	0.003	1.006–1.017 *
Native Hawaiian/Pacific Islander (%)	1.016	0.010	0.996–1.036

**Table 4:** Results of multivariate model of COVID-19 testing rates based on SES and people of color variables. Estimates and 95% CIs are the exponentiated coefficients of the Poisson model, with statistically significant associations indicated with an asterisk.

	Estimate	Robust SE	95% CI
Intercept	0.643	0.180	0.371–1.115
Low Educational Attainment (%)	1.005	0.004	0.998–1.013
People of Color (%)	0.994	0.002	0.990–0.998 *
Unemployment (%)	0.993	0.010	0.974–1.013
Transportation Cost (%)	0.915	0.012	0.892–0.938 *

**Table 5:** Results of multivariate model of COVID-19 testing based on SES and race/ethnicity variables. Estimates and 95% CIs are the exponentiated coefficients of the Poisson model, with statistically significant associations indicated with an asterisk.

	Estimate	Robust SE	95% CI
Intercept	0.659	0.152	0.420–1.035
Low Educational Attainment (%)	1.010	0.004	1.001–1.018 *
Unemployment (%)	0.993	0.009	0.976–1.010
Transportation Cost (%)	0.918	0.010	0.897–0.938 *
Hispanic (%)	0.983	0.003	0.977–0.989 *
Black (%)	1.003	0.003	0.997–1.008
American Indian/Alaskan Native (%)	1.005	0.016	0.975–1.037
Asian (%)	0.988	0.001	0.985–0.991 *
Native Hawaiian/Pacific Islander (%)	0.983	0.007	0.969–0.997 *

## Supplementary Materials

The following are available online at https://www.mdpi.com/1660-4601/17/24/9516/s1, Figure S1: Geographically weighted regression results for test positivity, Figure S2: Geographically weighted regression results for testing rates.
