# Supplementary material for: Community-Level Factors Associated with COVID-19 Cases and Testing Equity in King County, Washington
Source: Int J Environ Res Public Health. 2020 Dec 18;17(24):9516. doi: 10.3390/ijerph17249516 (PMC7767300; doi:10.3390/ijerph17249516)

Predicted Positivity Rate

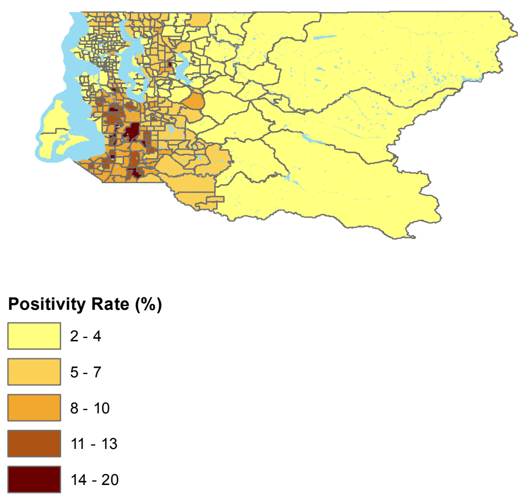

Deviance Residuals

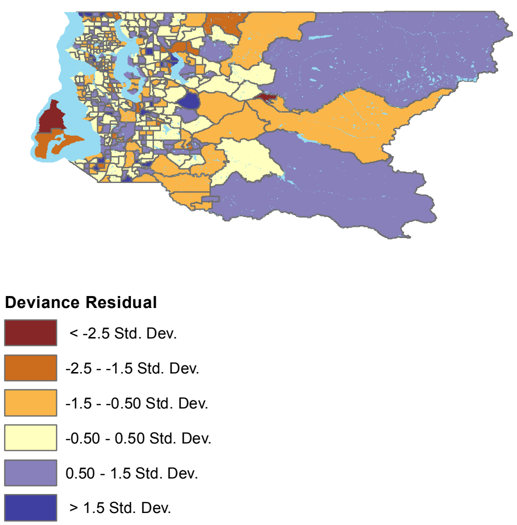

Low Educational Attainment

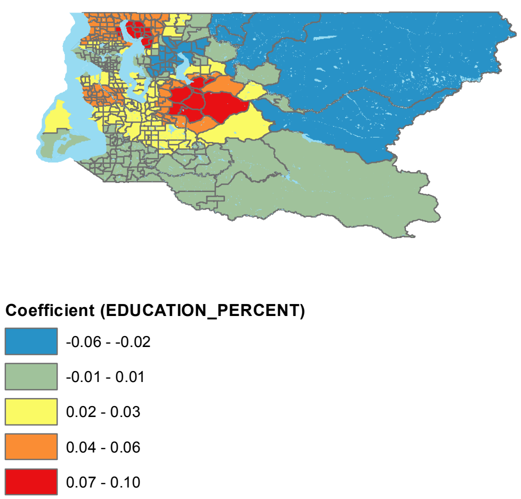

Poverty

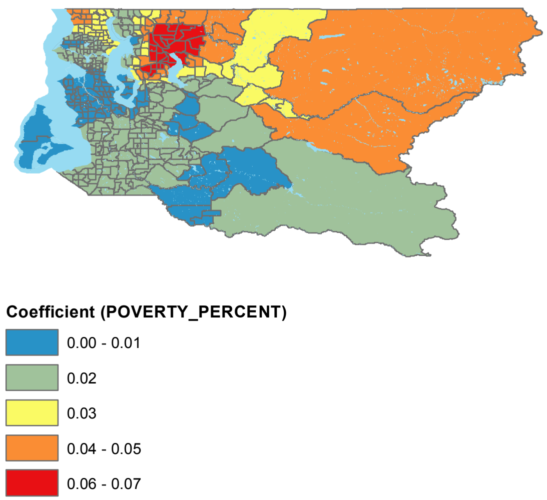

People of Color

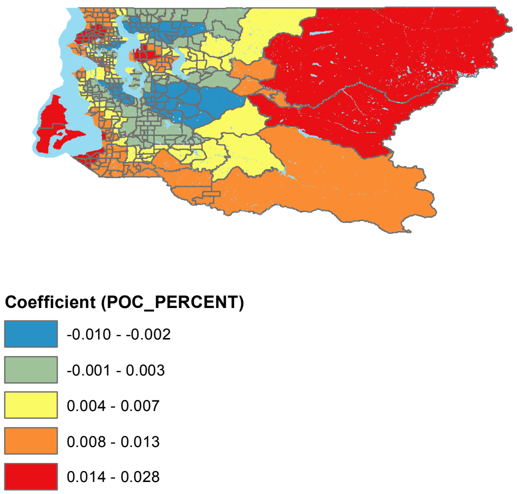

Transportation Cost

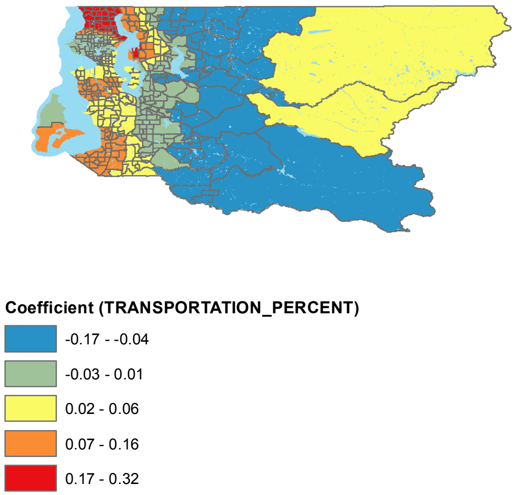

Supplement: Supplementary file 1 [file ijerph-17-09516-s001.zip › Figure S1_GWR_PositivityRate_maps.pdf]
