# Supplementary material for: Community-Level Factors Associated with COVID-19 Cases and Testing Equity in King County, Washington
Source: Int J Environ Res Public Health. 2020 Dec 18;17(24):9516. doi: 10.3390/ijerph17249516 (PMC7767300; doi:10.3390/ijerph17249516)
Supplement: Supplementary file 1 [file ijerph-17-09516-s001.zip › Figure S2_GWR_TestingRate_maps.pdf]

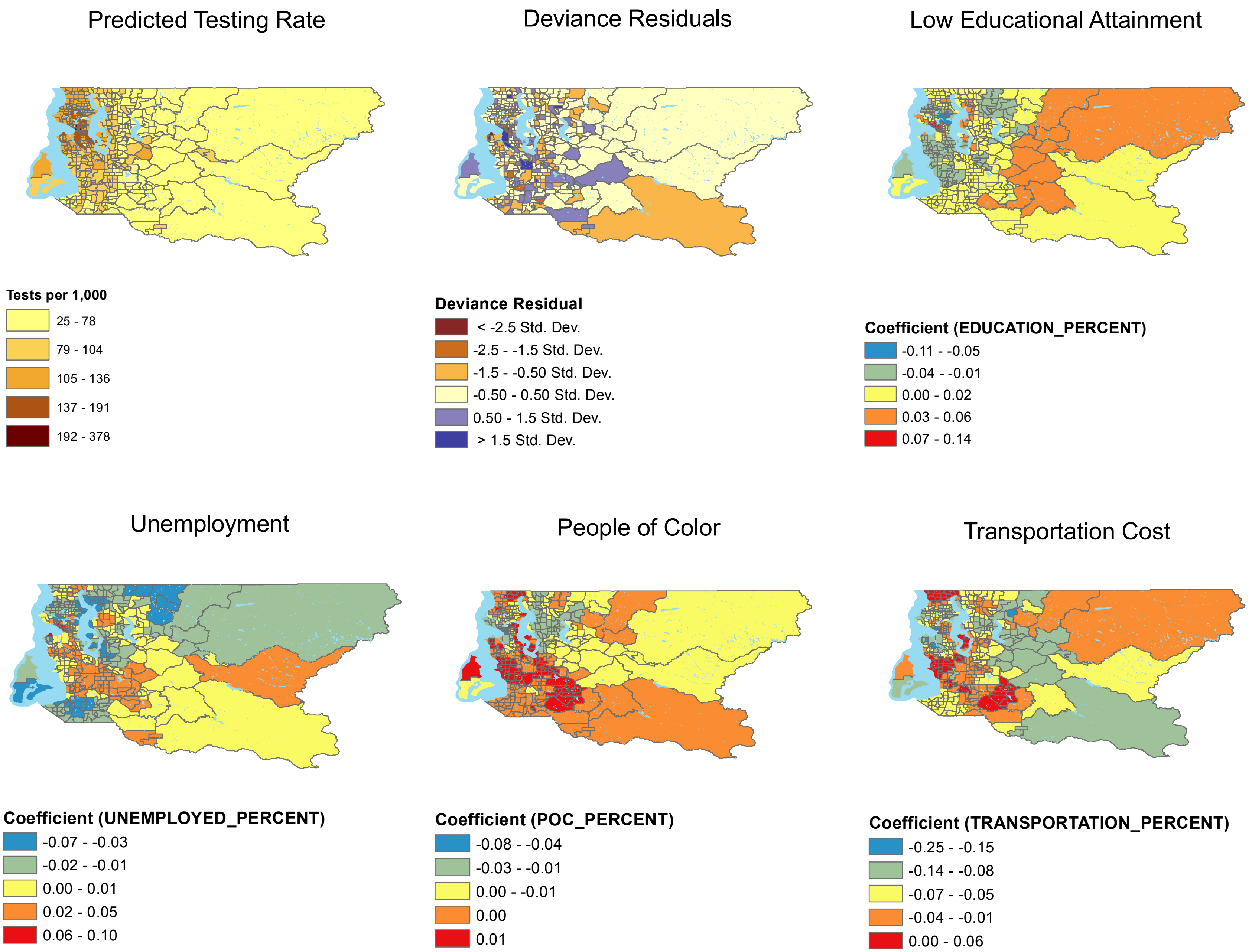
Predicted Testing Rate
Tests per 1,000
25 - 78
79 - 104
105 - 136
137 - 191
192 - 378
Deviance Residuals
Deviance Residual
< -2.5 Std. Dev.
-2.5 - -1.5 Std. Dev.
-1.5 - -0.50 Std. Dev.
-0.50 - 0.50 Std. Dev.
0.50 - 1.5 Std. Dev.
> 1.5 Std. Dev.
Low Educational Attainment
Coefficient (EDUCATION_PERCENT)
-0.11 - -0.05
-0.04 - -0.01
0.00 - 0.02
0.03 - 0.06
0.07 - 0.14
Unemployment
Coefficient (UNEMPLOYED_PERCENT)
-0.07 - -0.03
-0.02 - -0.01
0.00 - 0.01
0.02 - 0.05
0.06 - 0.10
People of Color
Coefficient (POC_PERCENT)
-0.08 - -0.04
-0.03 - -0.01
0.00 - -0.01
0.00
0.01
Transportation Cost
Coefficient (TRANSPORTATION_PERCENT)
-0.25 - -0.15
-0.14 - -0.08
-0.07 - -0.05
-0.04 - -0.01
0.00 - 0.06
